# Yeasts in nanotechnology-enabled oral vaccine and gene delivery

**DOI:** 10.1080/21655979.2021.1985816

**Published:** 2021-10-27

**Authors:** Elena Ivanova

**Affiliations:** Department of Biomedical Engineering, Columbia University, New York, NY, USA

**Keywords:** Oral vaccines, synthetic yeast, gene delivery, nanoparticles

## Abstract

Oral vaccine and gene delivery systems must be engineered to withstand several different physiological environments, such as those present in the oral cavity, stomach, and jejunum, each of which exhibits varying pH levels and enzyme distributions. Additionally, these systems must be designed to ensure appropriate gastrointestinal absorption and tissue/cellular targeting properties. Yeasts-based delivery vehicles are excellent candidates for oral vaccine and oral gene therapies as many species possess cellular characteristics resulting in enhanced resistance to the harsh gastrointestinal (GI) environment and facilitated passage across the mucosal barrier. Yeast capsules can stimulate and modulate host immune responses, which is beneficial for vaccine efficacy. In addition, recombinant modification of yeasts to express cell penetrating proteins and injection mechanisms along with efficient cell adhering capabilities can potentially improve transfection rates of genetic material. In this literature review, we present evidence supporting the beneficial role yeast-based delivery systems can play in increasing the efficacy of oral administration of vaccines and gene therapies.

## Introduction

The *oral administration of therapeutic agents is preferred because it is convenient, pain free, less fear-inducing, and can be self-administered obviating the need for trained personnel*. In addition, undesired off-target effects due to systemic administration may be avoided. The oral route is the most common and preferred method of drug administration [1,2], and new strategies for oral delivery of vaccines and gene therapies are continuously evolving.

Oral vaccines are often preferred over traditional injection-based formulations because of increased safety and compliance, as well as simpler manufacturing requirements [3]. Additionally, *oral administration of vaccines has physiologic advantages due to its ability* to enable immunostimulation at both mucosal and systemic sites, allowing more widespread protection against infectious diseases.

In the biomedical genetics community, the greater emphasis on eliminating the causative factors underlying a disease rather than treating symptoms has led to the rapid development of approaches to gene therapy and gene delivery. *Oral gene delivery has the potential to treat diseases specific to the gastrointestinal tract (GIT) such as ulcerative colitis and cancer and may also have broader therapeutic effects for diseases such as Type 2 diabetes* [4]

**Figure 1.**
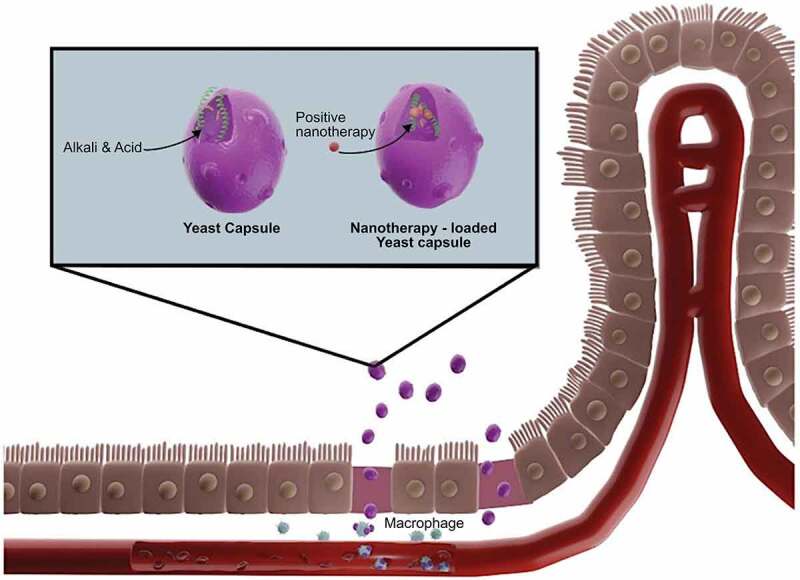
Nano therapies orally delivered via a yeast capsule (YC). Adapted from Zhang, X. et al., 2017

**Figure 2.**
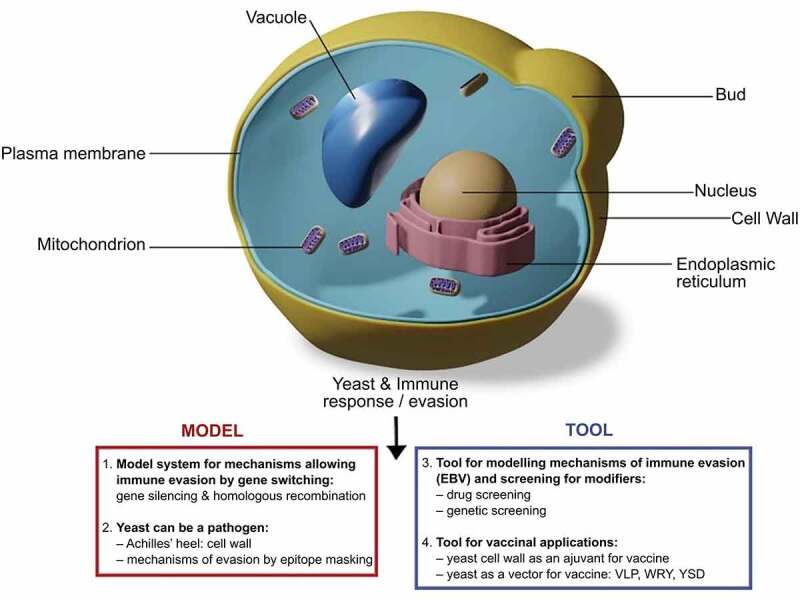
The various use of yeast to explore and modulate the immune response. Adapted from Angrand, G. et al., 2019

*However, the oral route presents distinct challenges and barriers. To gain access to the systemic circulation and reach the target tissue/cells, therapeutic agents must overcome a hostile GIT with varying pHs, enzyme degradation, a formidable mucous barrier*. In the case of pharmaceuticals, ~65% active pharmaceutical ingredient (API) loss is due to passage across GI mucosal barrier [1]. Additionally, drug availability in the circulation following oral intake has been shown to be approximately 25% of that attained with systemic injection [5]. Treatment availability is particularly important with vaccines and gene delivery systems because of their low transfection rates [6,7]. As vaccine development must be focused on inducing long-lasting immune responses, it is highly challenging to minimize any vaccine-associated side effects without compromising immunogenicity. In addition, oral vaccines face the additional challenge of undergoing hepatic first-pass metabolism and must therefore be designed to evade breakdown. For gene delivery, a high *in situ* transfection efficacy is one of the keys to success but must overcome potential interference caused by various physical and biochemical factors Figure 1 and 2.

An effective gene delivery system requires distinct engineering techniques that address safe and efficient transport of nucleic acid directly to target cells [8],and prevent metabolism and degradation [9]. Gene delivery systems must be designed for cell internalization, intracellular transport, and nuclear passage [10]. Given these requirements, gene delivery systems often show poor transfection efficiencies. Thus, the difficult task remains of how to effectively design oral gene delivery systems that combine all oral delivery requirements with the mandatory design specifications for gene deliver.

Yeasts are already commonly used in the commercial development and manufacturing of pharmaceutical and cosmetic products, particularly because of their ability to be engineered for mass bioproduction of various biological agents [11]. Yeasts possess unique characteristics that make them attractive vehicles for oral administration of vaccines and gene therapies. For example, yeasts’ glycocalyx-enabled adhesion properties have been shown to promote improved gastrointestinal passage and subsequent circulation of a wide array of active pharmaceutical compounds, enabling substantial advancements in therapeutic efficacy [12,13]. Yeast-derived microcapsules provide the proper protection for vaccine against the harsh GI environment as well as the suitable modality facilitating its transportation into the bloodstream [12,13]. Their ability to trigger a rapid immune response and amenability for genetic recombination/modification are among the advantages of yeasts as an oral delivery of vaccines and genes. In this literature review, we present current evidence supporting a role for using yeast-based delivery systems that can potentially overcome the current limitations of oral-based administration of therapeutic agents.

## Oral vaccines- current state

Many types of vaccines exist, including inactivated [14], live-attenuated [15], mRNA [16], DNA [17], protein [18], polysaccharide [19], conjugate [20], toxoid [21], and viral vector vaccines [22]. Most vaccine types have shown promising efficacy in an oral dosage form. Some examples include human insulin conjugated to cholera toxin was prepared as an oral vaccine that successfully suppressed beta cell destruction and diabetes in a diabetic mouse model [23]. In another study, Betanodavirus coat protein expressed in tobacco was tested as an oral vaccine in fish and showed significant protection against the virus [24]. Oral delivery of a DNA vaccine based on envelope proteins successfully induced high titers of the specific antibody against the tembusu virus in ducks [25]. Similarly, a typhoid vaccine composed of polysaccharides, inactivated influenza, live attenuated coronavirus, and adeno-associated virus vectors (AAV) all showed potential as oral vaccines in various animal models [25–29]. Despite the promising experimental outcomes, the above-listed forms of oral vaccines still remain in developmental stages and have rarely been tested in human trials, suggesting further improvements in delivery, transfection efficacy, availability, and transportation are needed.

## Oral gene delivery-current state

Genetic material can be delivered *in vivo* via viral, nonviral, or hybrid viral/nonviral systems. Both viral and nonviral gene delivery systems have shown efficacy via the oral route in research settings, though results from most studies in animal models have not been promising in clinical settings [30,31]. A previous study reported use of a nonviral vector comprising branched polyethylenimine on chitosan (CS-g-bPEI) to deliver the insulin gene [30]. The copolymer-based nonviral vector was designed to protect the plasmid from gastric acidic degradation and to facilitate transport across the gut epithelium. When orally administrated in diabetic mouse model, CS-g-bPEI with insulin plasmid DNA nanoparticles resulted in transgene expression for days, leading to protection of animals from hyperglycemia for more than 10 days, supporting the feasibility of nonviral oral gene delivery [30]. Viral vectors, such as lentivirus, have also shown potential in the oral gene delivery. Lentivirus vector encoding murine interleukin-10 (IL-10) showed promising results in suppressing the development and relapse of experimental murine colitis [31]. However, these studies are limited to small animal models and are in their early developmental stages. Despite the promising outcome in mouse models, clinical translation of such oral gene delivery requires a more sophisticated delivery modality that enables the protection and transportation of viral vectors through a long GI track with a more complex biophysical and chemical environment [12].

## Yeasts as oral vaccine and gene delivery agents

### Yeast characteristics

While yeasts are lumped together and considered a single homogeneous group of fungi, Saccharomycetes is classified at the class taxonomic level, and all yeasts are further classified into order, family, genus, and species levels resulting in a wide variety of yeast morphologies, characteristics, and functions. Numerous species of yeast have been utilized for bioproduction, which is based on individual properties suitable for each application. For recombinant protein production, *Saccharomyces cerevisiae (S. cerevisiae), Komagataella species, Kluyveromyces lactis (K. lactis*), and *Yarrowia lipolytica (Y. lipolutica)* have been widely employed [32–35], while *S. cerevisiae* has been predominantly used for producing yeast cell wall membrane micro-/nanoparticle for drug/gene delivery [25,32,35–39]. As a baker’s yeast in food grade, *S. cerevisiae* is considered safe and nontoxic to humans [40], having demonstrated low systemic toxicity in clinical trials as a vaccine delivery vehicle [41,42].

Yeasts are unicellular microorganisms that contain a chitin-dense cell wall surrounded by a mucilaginous glycocalyx, which enables yeast cells to form rapid and firm adhesions to many nearby substrates including host cell membranes [43]. Chitin (and its natural analog chitosan) is found in all yeasts and is considered a gastrointestinal absorption enhancer (particularly for hydrophilic drugs) because of its pH-responsive nature [44]. Chitosan is insoluble at neutral and alkaline pH. However, it forms as inorganic and organic acids with mucoadhesive properties at acidic pH that enhances paracellular permeability by modulating epithelial junctions [31,44]. Such pH-dependent properties, together with mucoadhesive features due to glycocalyx system, make chitosan a promising gastrointestinal absorption enhancer for orally administrated drugs and genes to be readily transported to the blood stream given the low intestinal pH and the abundance of gastric mucosa [31,44].

Accordingly, yeasts have been adopted to deliver many pharmaceutical agents, including small- and large-molecule drugs [45–47], genetic materials [48], and vaccines [49]. For example, an oral delivery of yeast glucan particles carrying methotrexate, an anti-inflammatory drug, was shown to be effective in treating mice with inflammatory bowel disease [50]. The study reported that drug-containing yeast glucan particles were internalized through macrophages, with subsequent down-regulation of macrophage-induced pro-inflammatory cytokines. Orally administrated yeast-derived microparticles were also successfully endocytosed by macrophages and maintained in atherosclerotic lesions, suggesting its potential as oral treatment for cardiovascular diseases [46].

In mouse and human cellular in vitro studies, DNA and mRNA internalized in yeast microparticles have also shown promising results in inducing immune responses through binding and endocytosis by macrophages. Another study demonstrated that a model antigen, ovalbumin (OVA), conjugated to the surface of yeast microparticles were readily recognized and internalized by dendritic cells [49]. Taken together, these findings consistently support a promising role for the utilization of yeasts as a highly efficient oral delivery vehicle for various types of drugs, genes and vaccines.

### Yeasts for oral vaccine delivery

Every vaccine aims to achieve a distinct, desired immune response. Encapsulation of the active components of the vaccine within yeast micro- or nanocapsules is one method for facilitating their use in oral delivery systems (ODSs). The yeast cell membrane (complete with the chitinous cell wall and glycocalyx components) can be transformed into a microencapsulation system (similar to a liposomal or exosomal shell) that can house and carry the desired vaccine cargo [48]. In a previous study, human dendritic cells were loaded with yeast-derived microparticles carrying DNA or mRNA of human pp65 that in turn stimulated peripheral blood lymphocytes leading to activation of CD8 memory cells [48]. As yeast-based systems do not require additional protein to release and/or target transduction of DNA/mRNA, it may represent a superior delivery system for many bacterial and viral systems in terms of efficacy, safety, and targeting [48].

Recombinant modification of yeasts can also be applied to enable genetic transfection of human cells for the production of viral proteins which represents another strategy for the application for developing orally administered mRNA or DNA vaccines [51]. Recombinant yeast strains were generated to contain IL-1β shRNA vector by transforming yeast with plasmids pIN27-hU6-shRNA-miR30 [52]. These recombinant yeasts downregulated IL-1β expression in macrophages, consequently leading to alleviation of the inflammation caused by osteoarthritis in a mouse model [52]. These findings support the advantage of yeast as a versatile tool to be readily modified and engineered for specific goals. However, there are still remaining challenges in the commercialization and mass production of yeast-based oral vaccine as an inactive, dry tablets [53].

Although yeasts can in theory be employed as delivery vehicles for inactivated or live-attenuated vaccines, they are better vehicles for delivering mRNA, DNA, protein, polysaccharide, toxoid, or certain viral vector vaccines because of the more manageable size requirements (< 100 nm). Certain vaccine types are more amenable to both oral administration and yeast delivery. Smaller (< 100 nm) active vaccine agents allow better engineering of the yeast capsule in terms of design and size, which means that yeasts should primarily be utilized for the oral delivery of mRNA, DNA, protein, polysaccharide, or toxoid vaccines, but only if systemic circulation is the end goal (i.e., additional targeting to peripheral tissues after access to the bloodstream is not required).

### Yeasts for oral gene delivery

The use of yeasts for gene delivery can be achieved through [1] enclosing genetic material in yeast-derived capsules [52] and [2] live recombinant yeast transfection [51]. With gene delivery, successful transport across the gastric mucosa and access to the circulation is only the first step in a complex series of pathways that include, but are not limited to, targeting of specific cells, cellular uptake of DNA, intracellular movement or trafficking of DNA, nuclear translocation and unpacking. While limited studies have demonstrated gene editing can be achieved in non-GI tissues following oral administration *in vivo* [39,54], transfection was relatively untargeted, and transfection efficiencies, when reported, were relatively low. For example, non-virus-mediated interleukin-1β (IL-1β) short hairpin RNA (shRNA) were orally delivered via yeast microcapsules in mice to mitigate progression of osteoarthritis [52]. The orally delivered IL-1β shRNA reduced IL-1β expressions not only in intestinal macrophages but also in bone marrow macrophages and articular cartilage, consequently leading to reduction of the joint inflammation [52]. This study suggests that oral gene delivery via yeast microcapsules may be a feasible gene therapy strategy for treating tissue-specific problems, through the mononuclear phagocyte system from the intestine to systemic circulation toward the target tissue.

The advantages offered by yeasts under these circumstances mirror those described for their use in oral vaccine delivery. For one, the ability of yeasts to adhere closely to target cells for an extended period increases transfection rates by allowing more time cellular uptake of DNA or RNA uptake [55]. In addition, certain types of yeast can be selected or undergo recombinant construction allowing for the elaboration injection mechanisms and/or membrane-penetrating proteins on their cell surface that further augment genetic transfection [56,57].

## The impact of yeasts on the immune system

Over the past two decades, there have been numerous studies investigating the use of yeasts in raising an immune response against different pathogenic species [58–60]. While certain pathogenic yeasts have developed immune-evading strategies, such as epitope-masking to control the production and exposure of highly antigenic cell wall proteins (*Candida albicans)* [58] or epigenetic switching to selectively silence their expression (*Plasmodium species, Pneumocystis species, and Trypanosoma*) [59], the high cell wall antigenicity other yeast such *Saccharomyces cerevisiae* make them prime candidates for the creation of vaccine adjuvants [60].

Several components of yeast capsules can stimulate or modulate the host immune response. The inner yeast cell wall comprises β-1,3-glucan (80–90%), β-1,6-glucan (8–18%), and chitin (1–2%), while the outer cell wall largely consists of mannoproteins [59]. β-glucans are immediately recognized by dectin-1 and complement receptor 3 (CR3) in the host body, resulting in subsequent opsonization, an influx of inflammatory cells, and T-cell responses (Th17, Th1, and cytotoxic). This initiation of multiple avenues of T-cell response is a promising alternative to antibody-biased (Th2-type) T-cell responses, leading to their widespread use as vaccine adjuvants.

β-glucans are not the only immune-inducing components present in yeast cells. Both mannan and chitin bind to pattern recognition receptors, which can induce characteristic Th1 or Th17 T-cell responses dependent on the ratio of mannan and chitin, through distinct opsonization protocols. Mannan elicits a strong pro-inflammatory cytokine release effect and can aid in dendritic cell maturation, essentially helping the body develop a more mature immune system [61].

The yeast cell wall compiles multiple highly antigenic components in a single location enabling a multi-pronged immune activation, namely through the recognition of β-glucans by dectin-1 and CR3 leading to activation of other inflammatory cells and T-cell responses [58,59,61]. The mucosal and systemic immunity triggered by oral vaccine delivery could be enhanced with the incorporation of yeasts, potentially leading to higher baseline antibody levels following fewer doses.

## Manufacturing and formulation of yeast-delivery systems in manufacturing of pharmaceuticals

In sporadic cases, whole-cell yeasts (either live, attenuated, or dead) serve as therapeutic delivery devices without modification. The majority of yeast-delivery vehicles, however, require preliminary manufacturing and formulation steps, as they simply utilize the yeast cell membrane and wall components to establish encapsulation systems. The crudest microencapsulation procedure involves stirring the active pharmaceutical ingredient (API) into a yeast-water slurry under mild heat (~40°C) to gently melt the yeast cell membrane [11]. Lipid material is stripped from the yeast cell membrane and organelles, self-assembling into a micelle-like capsule that holds the API. The yeast microcapsules can then be formulated into a powder by drying through either spraying, fluidized bed, or freeze-drying.

Unfortunately, this system has a clear disadvantage. First, the process results in heterogeneous loading, with a wide distribution of API molecules per capsule. Second, the method only functions with small-molecule drugs (>1000 Da) and can degrade nucleic acid-based APIs [11]. Third, the API must display at least moderately hydrophilic characteristics to allow for co-miscibility within the yeast-water slurry. Although yeasts are not yet commercially utilized as delivery agents in pharmaceuticals, their widespread prevalence in the industry points to a potentially straightforward route for their commercial use as delivery vehicles.

## Manufacturing and formulation of yeast-delivery systems for oral vaccine and gene delivery

While the standardized crude yeast encapsulation method may work for certain vaccine subtypes, such as toxoid or small polysaccharide vaccines, modified formulation protocols have been implemented for DNA or mRNA loading and encapsulation for enhanced protection and delivery to target cells [39,51]. Nanotubes, a new class of biomimetic supermolecules nanomaterials formed from self-assembled synthetic DNA, have shown the unique chemical and physical properties suitable to transport small-molecule RNA to cells and tissues with low toxicity, excellent biocompatibility and biodegradability [62]. In a mouse model, a nanotube-RNA delivery system based on yeast cell wall particles was used in the oral delivery of miR365 antagomir for the treatment of posttraumatic osteoarthritis [39]. Layer-by-layer fabrication technique has also shown potential for constructing a more advanced multilayered yeast cell wall microcapsule to delivery DNA via the oral route [51]. While such advanced formulation tricks have been accomplished [39,51,52], more commercially relevant approaches can be adapted from exosome preparation processes, such as utilizing proteins to induce biogenesis of extracellular vesicles [63,64] or an anti-viral signal [65].

Surprisingly, these methods have not been explored for gene delivery and there is scarce literature investigating these approaches. The most common method for loading RNA into yeast capsules is to genetically modify the yeast to produce the desired RNA strand and subsequently induce yeast capsule formation [54]. If simply utilizing individual yeast components instead of entire yeast-derived capsules is preferred, facile sonochemical method can ensure adequate RNA loading [65]. For instance, thiolated chitosan, instead of entire yeast cell wall capsule, has been used to form nanocapsules between 250 and 570 nm in diameter with simultaneous loading with RNA [65].

Yeasts provide a wide array of specialized formulations. First, yeasts can endow the delivery vehicle with unique properties that can be driven by a display of unique surface proteins through individualized glycocalyx engineering. Indeed, most yeasts can be recombinantly modified to render added non-native capabilities. Second, the size of the yeast capsule should be optimized to meet the size requirements of the cargo. In theory, yeast capsules can be designed large enough to encapsulate live or attenuated viruses or small enough to carry a single nucleic acid strand. The optimum size needed for the micro- or nanocapsule to be swallowed, traverse the esophagus, avoid GI metabolism, attach to the intestinal wall, or promote API passage, should be established.

## Disease targets for oral yeast systems

Because most yeasts trigger a rapid immune response, disease applications where treatment efficacy and immune stimulation can function synergistically are preferred [58–60]. Due to the low transfection efficiency, it is generally advised to restrict yeast-encapsulated gene-editing therapies to the GI tract alone [24,47,66]. Based on these recommendations, a few potential disease targets for yeast-delivered oral vaccines and oral gene therapies have been elucidated.

### Yeast-derived oral vaccine disease targets

#### Oral mRNA vaccines and influenza

For oral mRNA vaccines, one immediate (and very impactful) target disease is influenza. Since the development of current influenza vaccines requires months of design and engineering, influenza vaccines for each season must be based on predictions of the dominant influenza strains and arrangements made months in advance [67]. Unfortunately, these predictions are often incorrect [68]. In contrast, mRNA vaccines can be prepared rapidly – the new Moderna COVID-19 vaccine was synthesized in only 2 days [69].

Several research studies have suggested the strong potential efficacy of mRNA vaccines in combating the spread of influenza [70–72], and Moderna has already begun developing three mRNA-based influenza vaccines with clinical trials currently underway. However, each of these mRNA vaccines is not for oral administration because of nucleic acid metabolism and degradation in the GI tract. Using yeasts as the delivery vehicle could overcome this challenge to some degree by improving translocation across the gastric mucosa and increasing availability in the systemic circulation.

#### Oral mRNA vaccines and other diseases

Other strong disease candidates for oral mRNA vaccines include diseases caused by the Zika virus [72], rabies virus [70], Epstein–Barr virus [71], and various coronaviruses. Modified mRNA encoding premembrane M (prM), one of the three structural proteins of Zika virus, was prepared as encapsulated in lipid nanoparticles [72]. The preM mRNA vaccine resulted in protection against Zika infection in female mice as well as their fetuses [55]. Prophylactic mRNA-based vaccine encoding rabies virus glycoprotein also demonstrated efficacy in providing protection against rabies virus in a phase I clinical trial [70]. For Epstein–Barr (EBV) virus, a developing mRNA vaccine encoding five EBV glycoproteins (gp350, gH/gL/gp42, and gB) by Moderna Therapeutics has shown potential for reducing the rate of the virus-associated infectious mononucleosis. In addition to these examples, any viral disease that could be prevented with administration of mRNA vaccine therapy that leads to nonspecific immune stimulation could become a reasonable candidate for yeast-based oral delivery.

#### Oral DNA vaccines

Since DNA vaccines require transfection of a targeted subset of cells, oral delivery is frequently considered for direct targeting of the gastric mucosa [2,12,17,25]. There are few diseases appropriate for yeast-derived oral DNA vaccines, though some potential disease targets include bacteria- or virus-induced gastritis, ulcers, and stomach/intestinal cancers. In theory, current hepatitis B and acellular pertussis protein vaccines as well as the existing polysaccharide vaccines against pneumococcal and meningococcal diseases could be replaced with highly effective and possibly safer oral formulations based on yeast-delivery systems. The majority of these infections occur after the infectious agent crosses protective mucosal barriers such as that of the GI track. Therefore, an immunologically strong mucosal barrier should provide more efficient protection against disease [3]. However, the current vaccines for hepatitis B and acellular pertussis, which are administered parenterally, are unable to stimulate a mucosal immune response. Thus, oral DNA vaccines via yeast-based delivery could be an attractive alternative to overcome this limitation [3,73].

### Yeast-derived oral gene delivery disease targets

Since oral gene delivery must presently be confined to the GI system, potential disease targets for DNA vaccines include bacterial and viral gastritis, ulcers, and GI cancers. Genetic diseases of the digestive system, such as cystic fibrosis, Crohn’s disease, or Type 1 diabetes are also candidates for oral gene delivery [74,75]. For example, vaccines against the cystic fibrosis pathogen, *P. aeruginosa flagella*, have been developed in various forms targeting its fusion proteins; furthermore, recently developed nasal and oral vaccinations resulted in airway immunogenicity against the pathogen with superior efficacy compared to systemic vaccination [75].

Type 1 diabetes is a metabolic disease initiated by the autoimmune destruction of pancreatic insulin-producing beta cells and accompanied by the development of antigen-specific antibodies and cytotoxic T lymphocytes. Consequently, vaccination with diabetic autoantigens have been investigated as protective therapy [74]. In a mouse model, an oral vaccine constructed with live attenuated Salmonella for simultaneous delivery of autoantigens in conjunction with immunomodulatory cytokine genes to immune cells in the gut mucosa resulted in significant reduction of the development of diabetes [74]. Thus, the efficacy of these vaccines in primarily targeting GI mucosa will likely be further enhanced when incorporated with yeast-based delivery system.

## Conclusion

Yeasts represent a powerful tool that can bolster the transition of vaccine and gene delivery systems toward the oral route in the next few decades. To that end, yeast-delivery systems must be engineered to capitalize one their natural cellular adhesion and adjuvant properties. To achieve widespread adoption, yeast-derived ODSs must demonstrate enhanced protection of the active cargo, improved translocation across the gastric mucosa, and superior access to the bloodstream.

## Future directions

Yeasts are likely to be utilized in the commercial vaccine and gene delivery industry over the coming years, especially for delivery of oral mRNA vaccines. Initially, standard yeasts with commonplace manufacturing protocols are likely to be employed, with wider and broader applications soon to follow. One primary method is genetic engineering of the yeasts to display distinct antigens on their capsule surface (for vaccines), mucosa-penetrating epitopes, or gene-injection mechanisms [57]. However, systematic physicochemical characterization and size optimization of yeast capsules need to be accomplished prior to the widespread adoption of these techniques, as current research in this area is scarce.

Additionally, yeast capsules will likely be altered to create vaccines against yeast-based diseases such as candidiasis, balanitis, and vaginal yeast infection. However, such a surprisingly simple idea that has yet to be fully explored. Nanomaterial science may help optimize delivery via multiplex and multilayered nanoparticle systems; however, these systems often face commercialization hurdles. Better options may include a combination of multiple yeast types for synergistic co-stimulation of the immune or delivery mechanisms, which could be easily combined during the manufacturing process. If mucosal passage can be sufficiently bolstered via yeast encapsulation, the yeast capsules can be loaded with additional targeting nanoparticles to accurately target distinct tissues outside of the GI tract. In essence, the future is bright for yeast-based delivery, yet initial obstacles must be surmounted before they enter the mainstream.
